# Research on the Improvement of the Signal Time Delay Estimation Method of Acoustic Positioning for Anti-Low Altitude UAVs

**DOI:** 10.3390/s25092735

**Published:** 2025-04-25

**Authors:** Miao Liu, Jiyan Yu, Zhengpeng Yang

**Affiliations:** School of Mechanical Engineering, Nanjing University of Science and Technology, Nanjing 210094, China; lm01232025@163.com (M.L.); yzp_njust@njust.edu.cn (Z.Y.)

**Keywords:** anti-drone security, drone acoustic signal, time delay estimation, weighting function, generalized cubic cross-correlation, low signal-to-noise ratio

## Abstract

With the popularity of low-altitude small unmanned aerial vehicles (UAVs), UAVs are often used to take candid photos or even carry out malicious attacks. Acoustic detection can be used to locate UAVs in order to prevent malicious attacks by UAVs. Aiming at the problem of a large error in the time delay estimation algorithm under a low SNR, a time delay estimation algorithm based on an improved weighted function combined with a generalized cubic cross-correlation is introduced. By analyzing and comparing the performance of generalized cross-correlation time delay estimation of different traditional weighting functions, an improved weighting function that combines improved smooth coherent transform (SCOT) and phase transform (PHAT) is proposed. Compared with the traditional generalized cross-correlation weighted function, the improved weighted function has a sharper and higher peak value, and the time delay estimation error is smaller at a low SNR. Secondly, by combining the improved weight function with the generalized cubic cross-correlation, the main peak value is further increased and sharpened, and the time delay estimation performance is better than that when combined with the generalized cubic cross-correlation and the generalized quadratic correlation. Experimental results show that in complex outdoor scenes, the positioning error of the unimproved GCC PHAT method is 45.22 cm, and the positioning error of the improved weighted function generalized cubic cross-correlation algorithm is no more than 22.1 cm. Compared with the unimproved GCC PHAT method, the performance is improved by 35.55%. It is proven that this method is helpful for improving the positioning ability of low-flying UAVs and can provide help for anti-terrorism security against malicious attacks by UAVs.

## 1. Introduction

With the popularity of small UAVs, UAVs are sometimes used by terrorists for candid photography, reconnaissance, and even terrorist attacks. Therefore, it is of great significance to give early warning of the harassment by small malicious UAVs. Low-cost detection of small UAVs can be conducted by acoustic positioning.

Among the UAV positioning technologies in recent years, acoustic positioning is a relatively common and rapidly developing method [1]. It locates the position of the UAV by using the time difference between the sound generated by the rotor sliding air to reach different sensors when the UAV is flying [2]. The UAV acoustic location algorithm based on time difference of arrival (TDOA) is widely used because of its high positioning accuracy and low computational complexity. The positioning accuracy of TDOA is closely related to the time delay difference, array error, and array deployment mode [3], so the time delay estimation error has received extensive attention.

At present, in the study of delay estimation, the cross-correlation algorithm is simple to implement and can provide quite accurate time delay estimation for signals with sufficient SNR, which is widely used in the field of acoustic localization [4,5]. Based on the cross-correlation algorithm, Zhu Chao et al. analyzed and compared the delay estimation effect of the generalized cross-correlation algorithm with various traditional weighting functions, such as ROTH, SCOT, PHAT, and HB [6]. Due to the limited time delay estimation effect of the traditional weighting function and generalized cross-correlation algorithm at a low SNR, many scholars have studied various improved weighting functions [7,8,9,10]. Dou Huijing et al. proposed a generalized quadratic cross-correlation based on generalized cross-correlation and quadratic correlation to improve the accuracy of time difference estimation [11]. Li Dahua et al. proposed that the generalized cubic cross-correlation algorithm of the Eckart weighted method has better anti-noise performance than other algorithms under strong noise. However, the algorithm requires additional frequency domain analysis of the signal, which increases the complexity of the algorithm. The accuracy of its time delay estimation largely depends on the understanding of the signal propagation medium and accurate identification of the transmission function of the propagation medium, and as such, the algorithm requires a large amount of computation [12].

Based on the advantages of SCOT and PHAT weighting functions, this paper studies the best joint weighting form of SCOT and PHAT weighting functions at a low SNR. Combined with the advantages of the improved weighting function and the generalized cubic cross-correlation, the robustness of the algorithm at a low SNR is further improved. This method improves the accuracy of time delay estimation and anti-noise performance to a great extent, without the algorithm requiring a large amount of computation.

## 2. Theory and Methodology

### 2.1. Generalized Cross-Correlation Time Delay Estimation

Generalized cross-correlation time delay estimation involves calculating the weighted cross-power spectral density of two signals in the frequency domain and then converting it back to the time domain to obtain the weighted cross-correlation function to determine the time delay between the two signals [13].

Assume that the source signal is $s\left(t\right)$, and the signals received by the two microphones are $x_{1}\left(t\right)$ and $x_{2}\left(t\right)$, respectively. The noise is $n_{1}\left(t\right)$, $n_{2}\left(t\right)$; The time delay of the signal is $d\left(t\right)$; the amplitude of the signal received by the two microphones is $A_{1}\left(t\right)$, $A_{2}\left(t\right)$. Assume that the received signal model is:

$$\left\{\begin{array}{l} x_{1}\left(t\right)=A_{1}s\left(t\right)+n_{1}\left(t\right)\\ x_{2}\left(t\right)=A_{2}s\left(t-d\right)+n_{2}\left(t\right) \end{array}\right.$$

The cross-correlation function of the two received signals $x_{1}\left(t\right)$ and $x_{2}\left(t\right)$ is:

$$\begin{array}{cc} R_{x_{1}x_{2}}\left(\tau \right) & =E\left[x_{1}\left(t\right)x_{2}\left(t-\tau \right)\right]\\ & =E\left\{\left[A_{1}s\left(t\right)+n_{1}\left(t\right)\right]\left[A_{2}s\left(t-d-\tau \right)+n_{2}\left(t-\tau \right)\right]\right\}\\ & =A_{1}A_{2}R_{ss}\left(\tau -d\right)+A_{1}R_{sn_{2}}\left(\tau \right)+A_{2}R_{sn_{1}}\left(\tau -d\right)+A_{1}A_{2}R_{n_{1}n_{2}}\left(\tau \right) \end{array}$$

Assuming that $n_{1}\left(t\right)$ and $n_{2}\left(t\right)$ are stationary and uncorrelated Gaussian white noise independent of $s\left(t\right)$, the cross-correlation function of the two noises $n_{1}\left(t\right)$ and $n_{2}\left(t\right)$ and the signal $s\left(t\right)$ is:

$$R_{sn_{1}}\left(\tau -d\right)=E\left[n_{1}\left(t\right)\cdot s\left(t-d-\tau \right)\right]=0$$

$$R_{sn_{2}}\left(\tau \right)=E\left[n_{2}\left(t-\tau \right)\cdot s\left(t\right)\right]=0$$

The cross-correlation function of noise $n_{1}\left(t\right)$ and $n_{2}\left(t\right)$ is:

$$R_{n_{1}n_{2}}\left(\tau \right)=E\left[n_{1}\left(t\right)\cdot n_{2}\left(t-\tau \right)\right]=0$$

So, there is

$$R_{x_{1}x_{2}}\left(\tau \right)=A_{1}A_{2}R_{ss}\left(\tau -d\right)$$

$R_{ss}\left(\tau -d\right)$ Is the autocorrelation function of the source signal, and according to its property, $R_{ss}\left(\tau -d\right)\leq R_{ss}\left(0\right)$. That is, when τ−d=0, the correlation function $R_{x_{1}x_{2}}\left(\tau \right)$ reaches its peak, and the correlation between the signals received by the primary and secondary observatories is the largest, so the estimated value of the TDOA is $\hat{d}=arg\;\max\left\{R_{ss}\left(\tau -d\right)\right\}$ [14]. A cross-correlation function is defined as:

$$R_{x_{1}x_{2}}\left(\tau \right)=\frac{1}{2\pi }\int_{-\infty }^{+\infty }G_{x_{1}x_{2}}\left(\omega \right)e^{j\omega \tau }d\omega$$

where $G_{x_{1}x_{2}}\left(\omega \right)$ is the cross-power spectrum of signals $x_{1}\left(t\right)$ and $x_{2}\left(t\right)$.

Generalized cross-correlation is developed based on basic cross-correlation, and its essence is to introduce a weighted function to filter the cross-power spectral density function, thereby enhancing the target signal, reducing the influence of noise, and making the peak value of $R_{x_{1}x_{2}}\left(\tau \right)$ larger and sharper [15]. The generalized cross-correlation function is defined as:

$$R_{x_{1}x_{2}}\left(\tau \right)=\frac{1}{2\pi }\int_{-\infty }^{+\infty }\phi_{x_{1}x_{2}}\left(\omega \right)G_{x_{1}x_{2}}\left(\omega \right)e^{j\omega \tau }d\omega$$

For different target signalls and environment noise, the weighting function is also different.

### 2.2. Analysis and Improvement of Weighting Function

Several common weighting functions are shown in Table 1.

The basic cross-correlation function is easily affected by noise, resulting in large time delay estimation errors. The ROTH weighting function has a certain suppression effect on noise, but it broadens the peak value. The SCOT weighting function considers the signals of two channels at the same time, but if the power spectral density of the signals of two channels is equal, the peak value of the correlation function will be broadened, and the accuracy of the time delay estimation will be reduced. The PHAT weighting function has a high time delay estimation accuracy for signals with large SNR, which is suitable for broadband signals, while it will produce large errors for signals with low power [16]. Aiming at the problem of the low time delay estimation performance of the traditional weighting function mentioned above, this paper proposes an improved weighting function:

$$\phi \left(\omega \right)=\frac{\left|G_{x_{1}x_{2}}\left(\omega \right)\right|}{{\left[G_{x_{1}x_{1}}\left(\omega \right)G_{x_{2}x_{2}}\left(\omega \right)\right]}^{\alpha }+\beta }$$

The improved weighting function combines the characteristics of SCOT and PHAT, improves the SCOT function, adds an exponential factor *α* (0 < *α* < 1), and combines with the PHAT function to improve the ability of noise suppression and highlight the peak value of the correlation function. The weight function introduces the self-power spectrum function and cross-power spectrum function of the two signals, and the numerator and denominator change trend is the same, which is suitable for signals of different strengths. The value of *α* needs to be determined by several experiments. The constant *β* (*β* ≥ 0) is introduced so that the denominator of the weight function will not go to 0 when the signal energy is low, thus reducing the estimation error. For non-low-energy signals, the parameter *β* can be ignored and simplified as:

$$\phi \left(\omega \right)=\frac{\left|G_{x_{1}x_{2}}\left(\omega \right)\right|}{{\left[G_{x_{1}x_{1}}\left(\omega \right)G_{x_{2}x_{2}}\left(\omega \right)\right]}^{\alpha }}$$

### 2.3. Generalized Cubic Cross-Correlation Time Delay Estimation

Based on the cross-correlation between two channels, the quadratic cross-correlation algorithm integrates the signal autocorrelation and then carries out a cross-correlation operation to improve the anti-noise performance [17]. $R_{11}$ is the autocorrelation function of signal $x_{1}\left(t\right)$, $R_{22}$ is the autocorrelation function of signal $x_{2}\left(t\right)$, and $R_{12}$ is the cross-correlation function of signals $x_{1}\left(t\right)$ and $x_{2}\left(t\right)$. There is:

$$R_{11}\left(\tau \right)=E\left[x_{1}\left(t\right)x_{1}\left(t-\tau \right)\right]$$

$$R_{22}\left(\tau \right)=E\left[x_{2}\left(t\right)x_{2}\left(t-\tau \right)\right]$$

$$R_{12}\left(\tau \right)=E\left[x_{1}\left(t\right)x_{2}\left(t-\tau \right)\right]$$

The expression of the quadratic cross-correlation is:

$$R_{RR}\left(\tau \right)=E\left[R_{11}\left(t\right)R_{12}\left(t-\tau \right)\right]=\eta R_{RS}\left(\tau -d\right)+R_{RN}\left(\tau \right)$$

where $R_{NS}$ and $R_{RN}$ are quadratic correlation functions of signal and noise, respectively.

**Figure 1:**
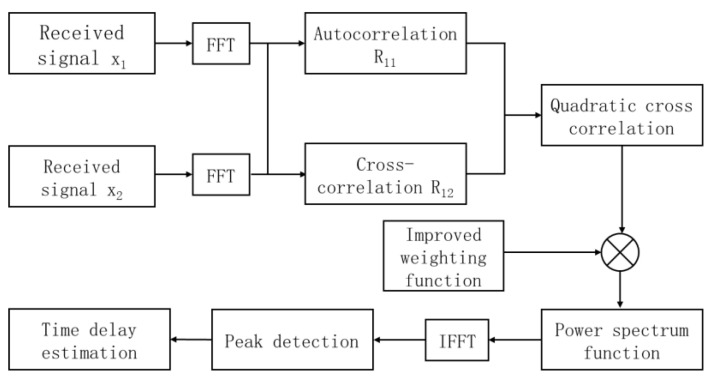
Flow chart of the improved weighted function joint generalized cubic cross-correlation algorithm.

In practical applications, the ambient noise is relatively complex, and certain errors will still occur at a low SNR. The threshold can be reduced by multiple cross-correlations of signals to improve the anti-noise performance of the algorithm [18]. On the basis of the quadratic cross-correlation operation, the cubic cross-correlation algorithm of two signals can be obtained. The cubic cross-correlation expression is:

$$R_{RRR}\left(\tau \right)=E\left[R_{22}\left(\tau \right)R_{R_{11}R_{12}}\left(t-\tau \right)\right]=\eta^{\prime }R_{sss}\left(\tau -d\right)+R_{nnn}\left(\tau \right)$$

where $R_{SSS}$ is the signal’s cubic cross-correlation, assuming noise is uncorrelated, then $R_{nnn}\left(\tau \right)=0$. When τ=d, the cubic cross-correlation function reaches the maximum value, and τ is the desired time delay value. The generalized cubic cross-correlation algorithm is used to obtain the power spectrum function by introducing the weighting function into the cubic cross-correlation function, converting it into the time domain using Fourier inversion, and finally detecting its peak value to obtain the estimated delay. The flow chart of the improved weighted function joint generalized cubic cross-correlation algorithm proposed in this paper is shown in Figure 1. Generalized cubic cross-correlation function expression:

$$R^{\prime }\left(\tau \right)=\frac{1}{2\pi }\int_{-\infty }^{+\infty }\phi \left(\omega \right)G_{RRR}\left(\omega \right)e^{j\omega \tau }d\omega$$

## 3. Analysis of the Sound Characteristics of UAVs

**Figure 2:**
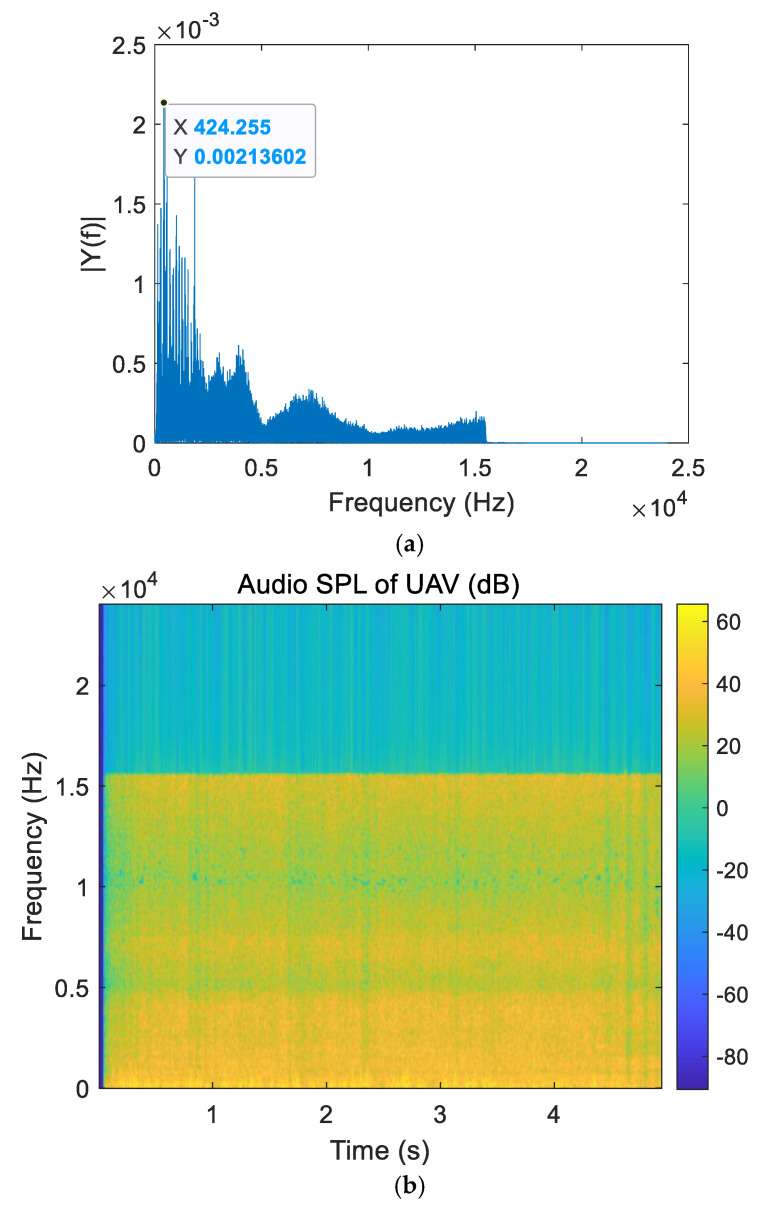
Spectrum analysis of UAV acoustic signals. (**a**) Frequency distribution of UAV acoustic signals. (**b**) Sound pressure level of UAV acoustic signals.

An audio clip of a drone flying at a height of 10 m was recorded in an outdoor experimental environment for spectrum analysis. The unilateral amplitude spectrum of the UAV acoustic signal is shown in Figure 2a. The sound pressure levels of different frequencies are shown in Figure 2b.

As can be seen from Figure 2, the main frequency band of the drone’s sound is concentrated between 130 and 5000 Hz, with the main frequency at 424.3 Hz. The audio in this frequency band is mainly generated by the propeller stirring the air, while the audio above 5000 Hz is produced by the friction of the internal components of the motor.

Based on the inverse square law in acoustics, given the sound pressure level at the reference distance, the sound pressure level of the UAV at different distances can be estimated. The calculation formula is:

$$L_{p}\left(r\right)=L_{p}\left(r_{0}\right)-20\log_{10}\left(\frac{r}{r_{0}}\right)$$

$L_{p}\left(r\right)$ is the sound pressure level (dB) at a distance of r, r is the target distance for calculating the sound pressure level, and $r_{0}$ is the reference distance of 10 m. The sound pressure level at 10 m was measured to be 62.35 dB.

**Figure 3:**
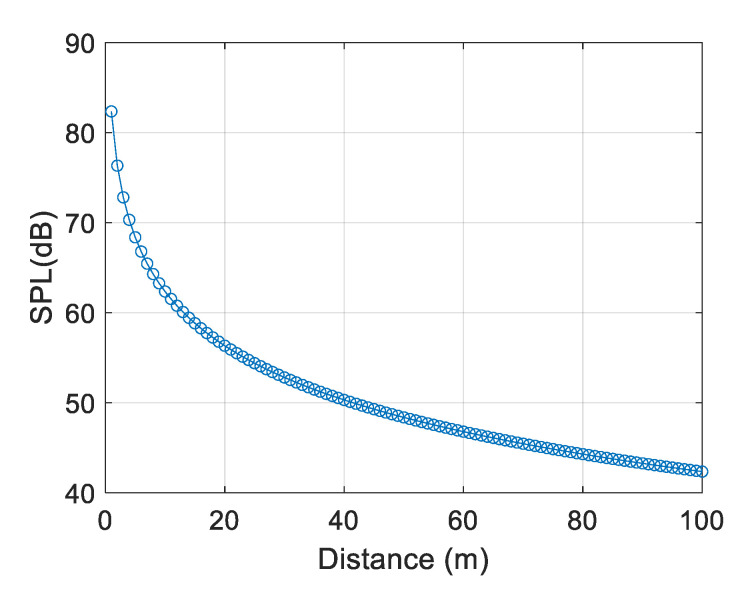
The sound pressure level of the UAV at different distances.

The sound pressure level of the unmanned aerial vehicle at a distance of 1 to 100 m was calculated using the above formula, as shown in Figure 3.

## 4. Simulation Analysis

### 4.1. Determination of the Value of α in the Improved Weighting Function

**Figure 4:**
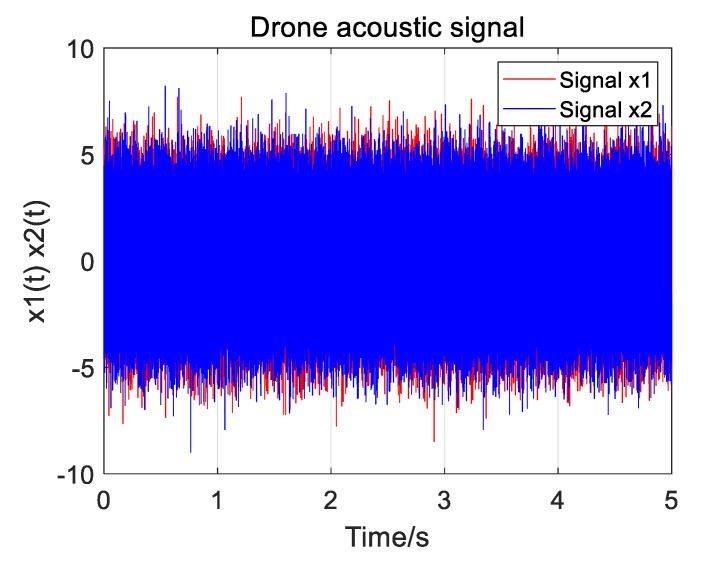
Two UAV sound signals.

**Figure 5:**
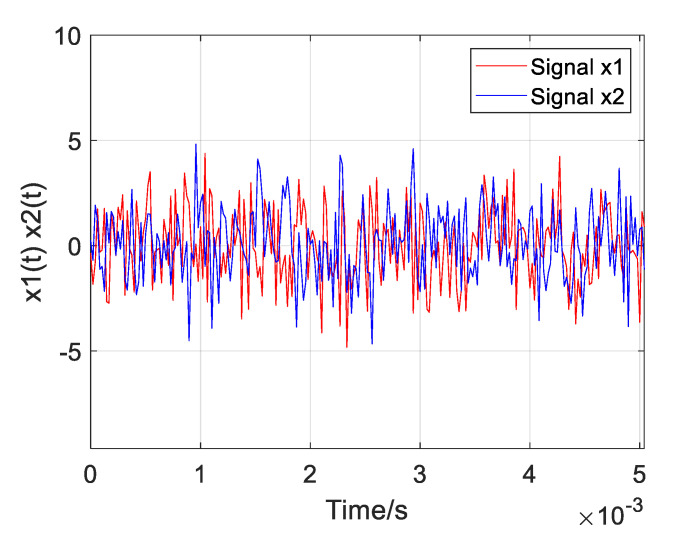
Two audio signals of the UAV after local amplification.

In order to determine the value of *α* in the improved weighting function, a recorded UAV sound signal is used for simulation research. The sampling rate is 48 kHz, and the time delay is set to 0.002 s. Stationary Gaussian white noise is added to the signal, and when the SNR is −5 dB, the two simulated received UAV signals are shown in Figure 4. The locally amplified acoustic signal of the unmanned aerial vehicle is shown in Figure 5.

**Figure 6:**
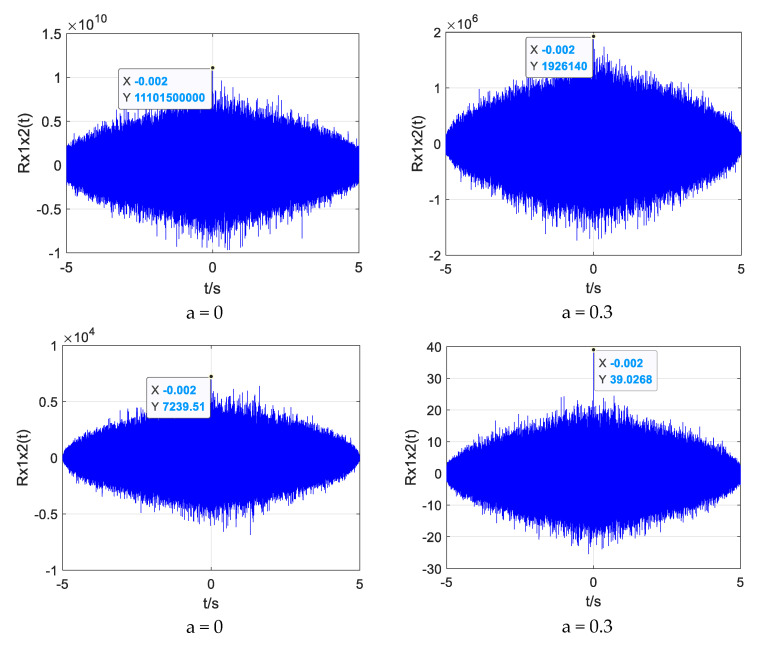

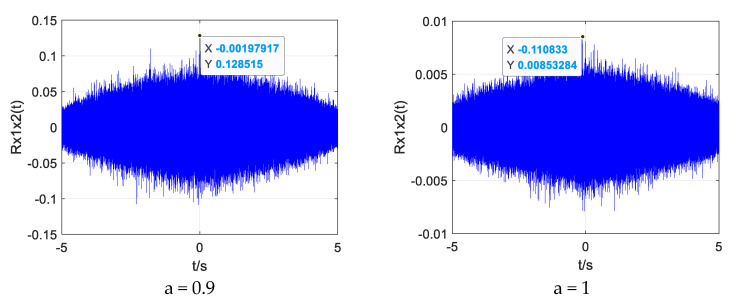
Time delay estimation results for different values of *α*.

Since the signal in Figure 2 is not a low-intensity signal, the *β* value is ignored during the simulation process. By changing the value of the exponential factor *α* many times, the value range of *α* is 0 < *α* < 1, and the peak value of time delay estimation is detected. The simulation results are shown in Figure 6.

From the above simulation results, it can be seen that the exponential factor *α* has an important effect on the time delay estimation performance. The correct time delay value can be obtained when *α* is less than or equal to 0.7, but the main peak is the most prominent when *α* is 0.7. When *α* is greater than 0.7, the spurious peak exceeds the correct peak, and the time delay cannot be estimated correctly. Therefore, when the sampling rate is 48 KHz, the delay estimation performance is the best when *α* is taken as 0.7. When the sampling rate changes, the value of *α* can be slightly adjusted according to the above steps to re-determine the optimal value of *α*.

### 4.2. Comparison of Generalized Cross-Correlation Algorithms of Different Weighted Functions

**Figure 7:**
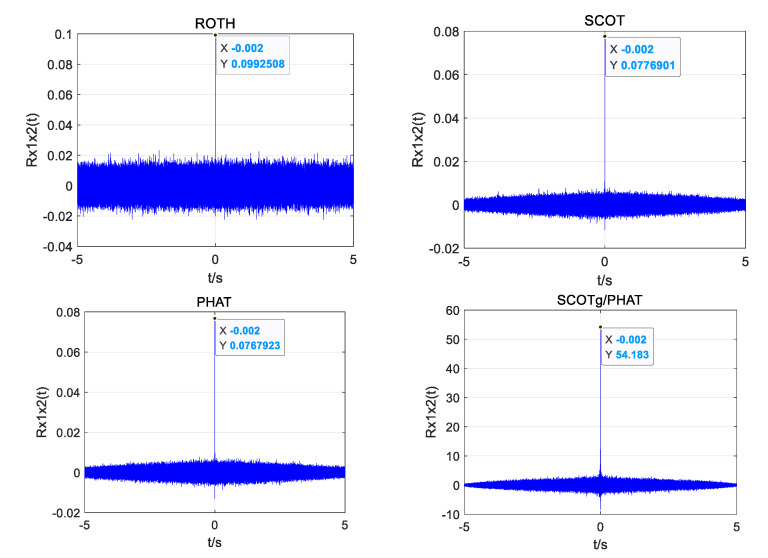
Simulation plots of four weighting functions at 10 dB SNR.

**Figure 8:**
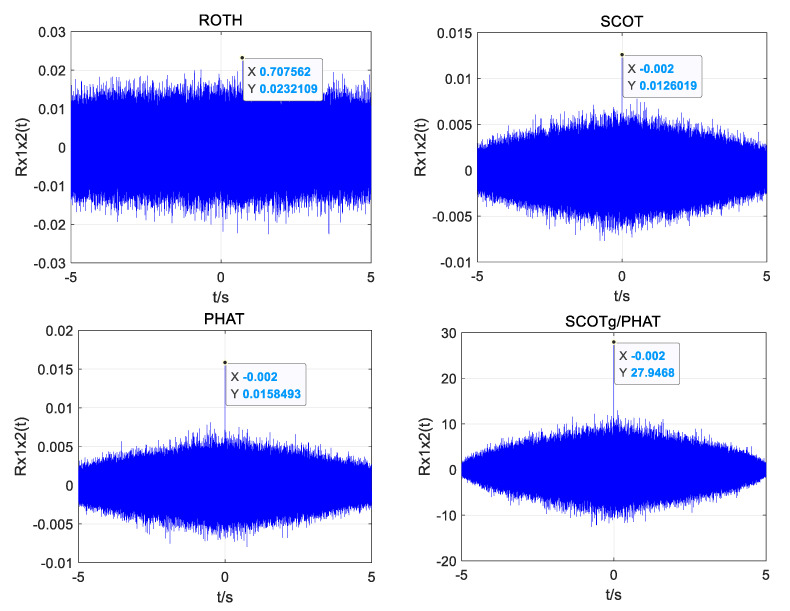
Simulation plots of four weighting functions at 0 dB SNR.

**Figure 9:**
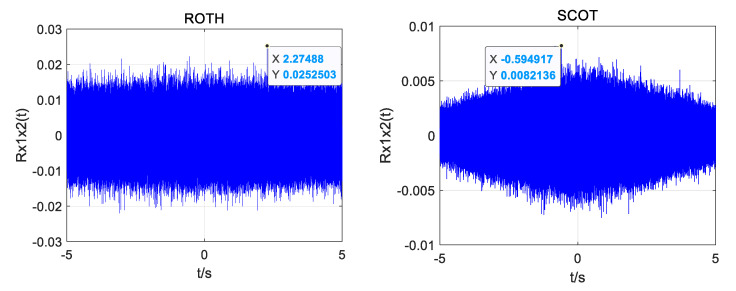

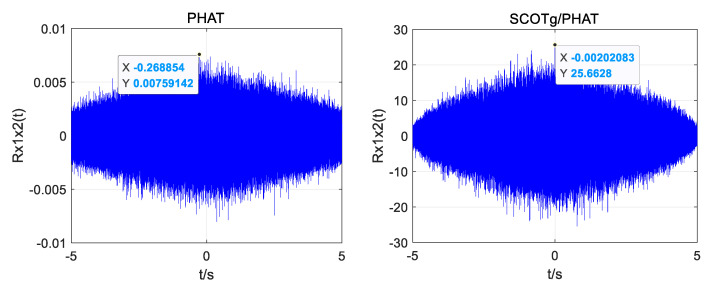
Simulation plots of four weighting functions at −5 dB SNR.

In order to compare and analyze the time delay estimation performance of different weighted functions under different SNR, ROTH, SCOT, PHAT, and improved weighted functions were used to compare the generalized cross-correlation time delay estimation for the same UAV sound signal. The index *α* of the improved weighting function is 0.7. The influence of different weight functions on the accuracy of time delay estimation under different SNRs was observed. Figure 7, Figure 8 and Figure 9 show the simulation results.

When the SNR is 10 dB, the four weighting functions can obtain the correct time delay estimation. However, the ROTH weighted generalized cross-correlation function has the largest number of false peaks, the SCOT and PHAT weighted functions have the same effect, and the modified weighted function has the highest peak and the smallest number of false peaks.

When the SNR drops to 0 dB, the ROTH weighted generalized cross-correlation algorithm has completely failed, and a large number of false peaks appear. Although the main peak value of SCOT, PHAT, and the improved weighting function is reduced, the correct time delay can still be obtained.

When the SNR drops to −5 dB, the generalized cross-correlation algorithms weighted by ROTH, SCOT, and PHAT all fail, and a large number of spurious peaks exceed the main peak. The time delay estimated by the generalized cross-correlation algorithm with improved weighting function also produces a little error, but it has certain anti-noise performance compared with other traditional weighting functions.

From the above simulation results, it can be seen that the main peak of the correlation function of the improved weighted function algorithm is less affected by the sub-peak, and the anti-noise interference ability is improved, showing good stability; however, there is also the phenomenon of sub-peak fluctuation.

### 4.3. A Comparison of the Improved Weighting Function of Different Joint Cross-Correlation Algorithms

**Figure 10:**
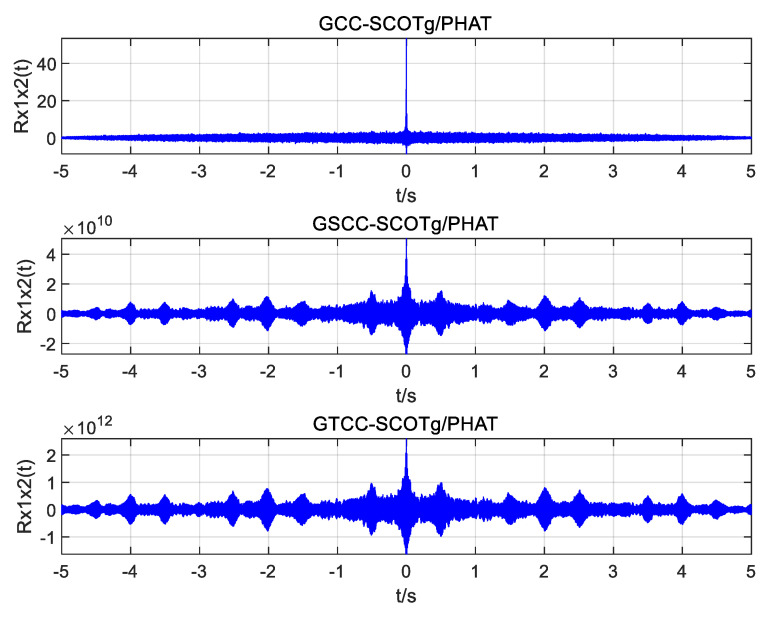
Simulation plots of the three algorithms with an SNR of 10 dB.

**Figure 11:**
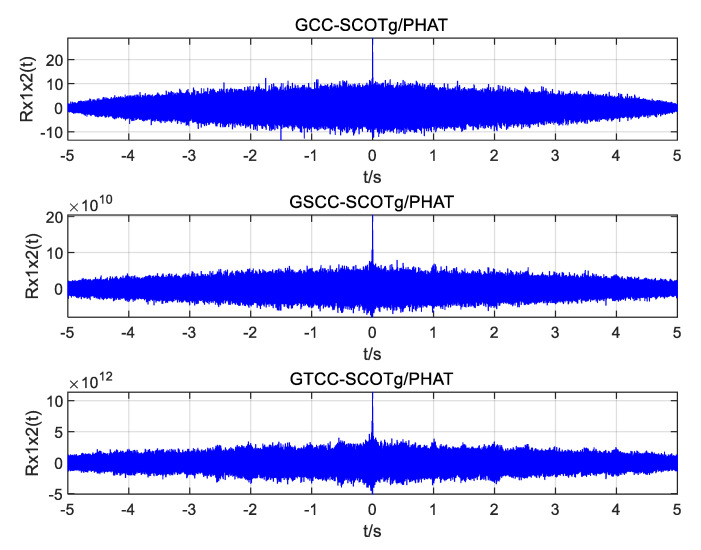
Simulation plots of the three algorithms with an SNR of 0 dB.

**Figure 12:**
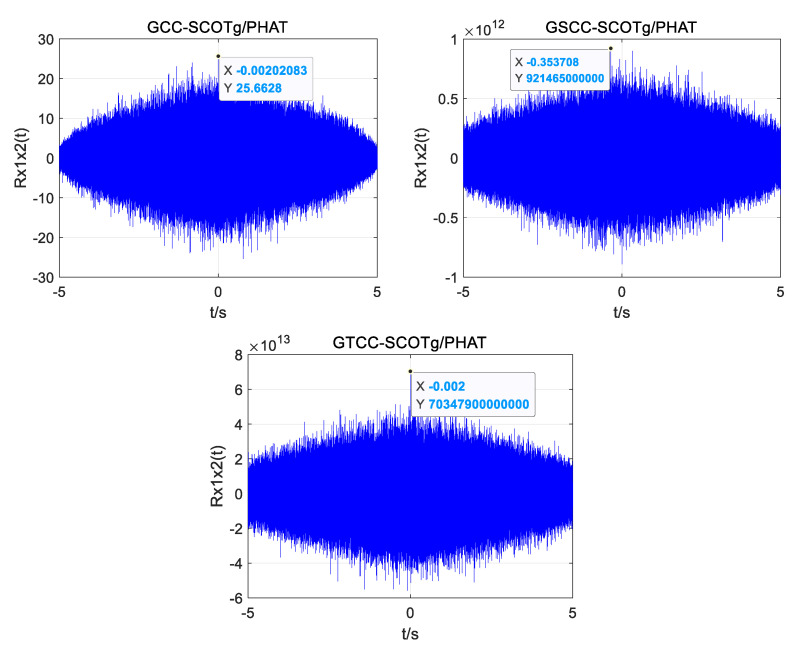
Simulation plots of the three algorithms with an SNR of −5 dB.

Using the above UAV sound signals, simulation experiments are carried out to improve the weighted function joint generalized cross-correlation (GCC), generalized quadratic cross-correlation (GSCC), and generalized cubic cross-correlation (GTCC). The time delay estimation performance of the three algorithms under different SNRs is also studied. Figure 10, Figure 11 and Figure 12 show the simulation results.

It can be seen from the simulation diagram that the three algorithms can obtain the correct time delay when the SNR is 10 dB and 0 dB, but the joint generalized cubic cross-correlation algorithm has the largest peak value and is much higher than the sidelobes. When the SNR is reduced to −5 dB, the spurious spectral peaks of generalized cross-correlation and generalized quadratic correlation exceed the correlation peaks, resulting in time delay estimation errors, but the correlation peaks are still obvious in the generalized cubic cross-correlation algorithm with improved weighted function, and it is easy to obtain the correct delay estimation.

In summary, when the signal-to-noise ratio is low, using the improved algorithm for time delay estimation can help obtain more accurate time delay estimation and has strong anti-noise performance.

### 4.4. Performance Comparison of Several Algorithms

**Figure 13:**
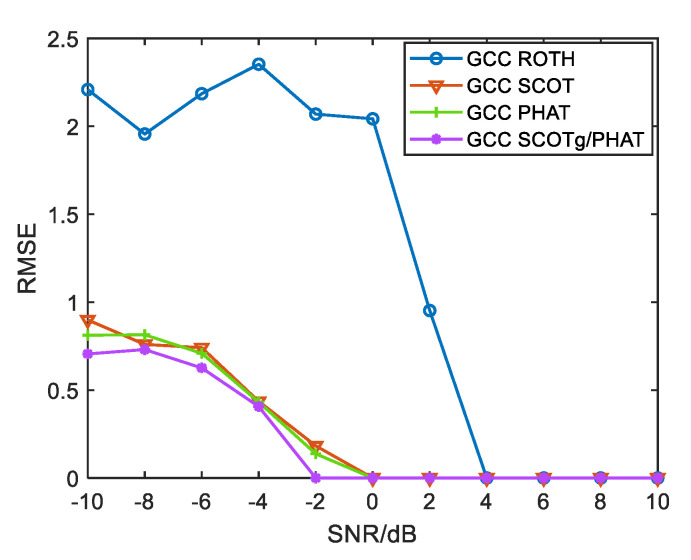
Time delay estimation error for each weighting function.

**Figure 14:**
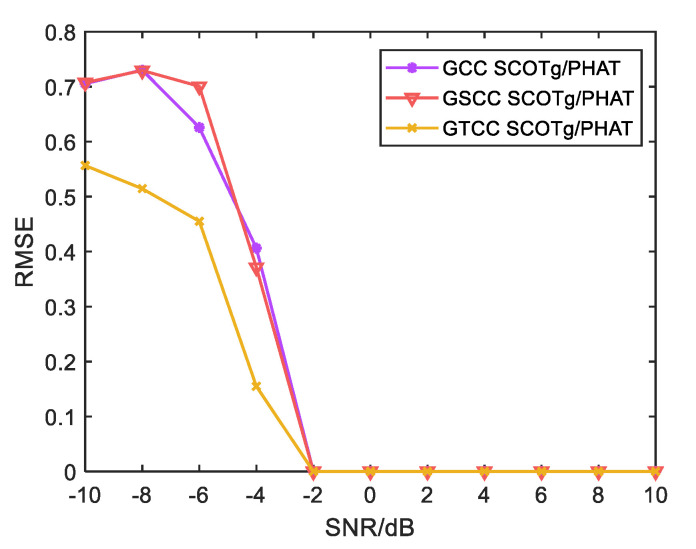
The comparison of a time delay estimation error for the improved weighted function combined with the first, second, and third cross-correlation algorithms.

In order to intuitively show the advantages of the improved weighted function compared with the traditional weighted function, and the advantages of the improved weighted function combined generalized cubic cross-correlation algorithm at a low SNR, this paper adopts the above algorithms for the time delay estimation of UAV sound signals. The signal-to-noise ratio is set to drop linearly from 10 dB to −10 dB, every 2 dB step. Fifty simulation tests were conducted under each fixed SNR, and the root-mean-square error was taken as the evaluation index. The results are shown in Figure 13 and Figure 14. The root-mean-square error is expressed as:

$$RMSE=\sqrt{\frac{1}{N}\sum_{i=1}^{N}{\left(\tau_{i}-\tau_{0}\right)}^{2}}$$

where, $\tau_{0}$ is the true time delay value, $\tau_{i}$ is the estimated time delay value obtained by the algorithm, and N is the number of time delays estimated by the algorithm.

As can be seen from Figure 13, the time delay estimation error of each weighting function gradually increases with the decrease in SNR. The performance of ROTH weighted time delay estimation is the worst. When the SNR is lower than 4 dB, the time delay estimation error increases sharply, and when the SNR is lower than 0 dB, the algorithm fails completely. The time delay estimation errors of SCOT and PHAT weighted algorithms are similar, and an accurate time delay can be obtained above 0 dB. The time delay estimation accuracy of the improved weighted function algorithm starts to decrease when the SNR is lower than −2 dB. Compared with other traditional weighted functions, the time delay estimation error of the improved weighted function is the smallest.

As can be seen from Figure 14, when the SNR is greater than −2 dB, the error of the three algorithms is almost 0, showing good time delay estimation performance. When the SNR is less than −2 dB, the time delay estimation errors of the three algorithms increase with the decrease in SNR. The generalized cubic cross-correlation algorithm with the improved combination of weighted functions has the smallest time delay estimation error among the three algorithms, which further improves the accuracy of the algorithm’s time delay estimation at a low SNR.

## 5. Experimental Verification

**Figure 15:**
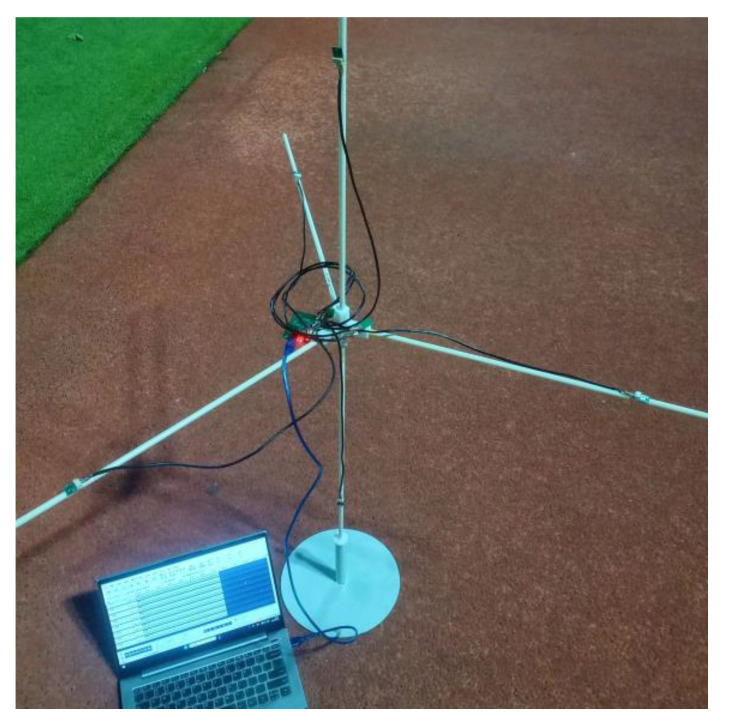
Five-element microphone array.

**Figure 16:**
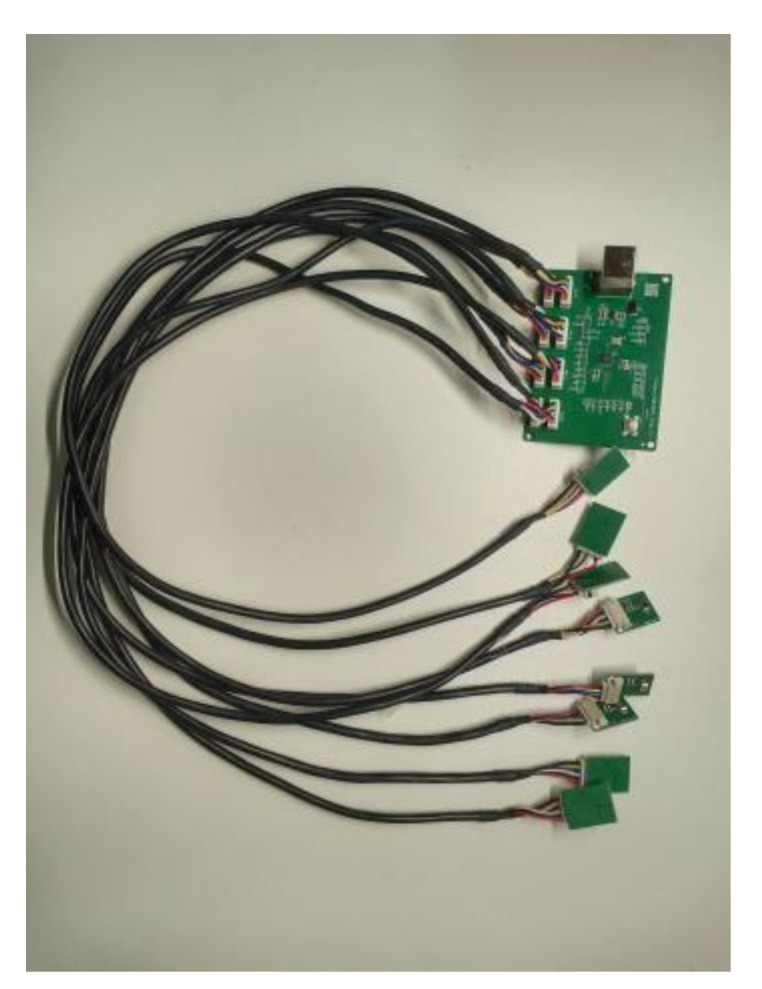
Microphone acquisition device.

In order to verify the performance of the time delay estimation algorithm, we used the five-element microphone array shown in Figure 15 to collect the UAV sound signal and conduct the time delay estimation test. The sampling frequency of each microphone is 48,000 Hz, and the array spacing of the microphone is 63.64 cm. The microphone device is shown in Figure 16. Table 2 shows the performance parameters of the microphone equipment. This device can simultaneously record data from eight microphones. The microphone type is a silicon microphone. The device can be arranged into different microphone arrays as required. The simulation and calculation are accomplished through MATLAB (Version:R2021b, created by MathWorks in Natick, MA, USA).

**Figure 17:**
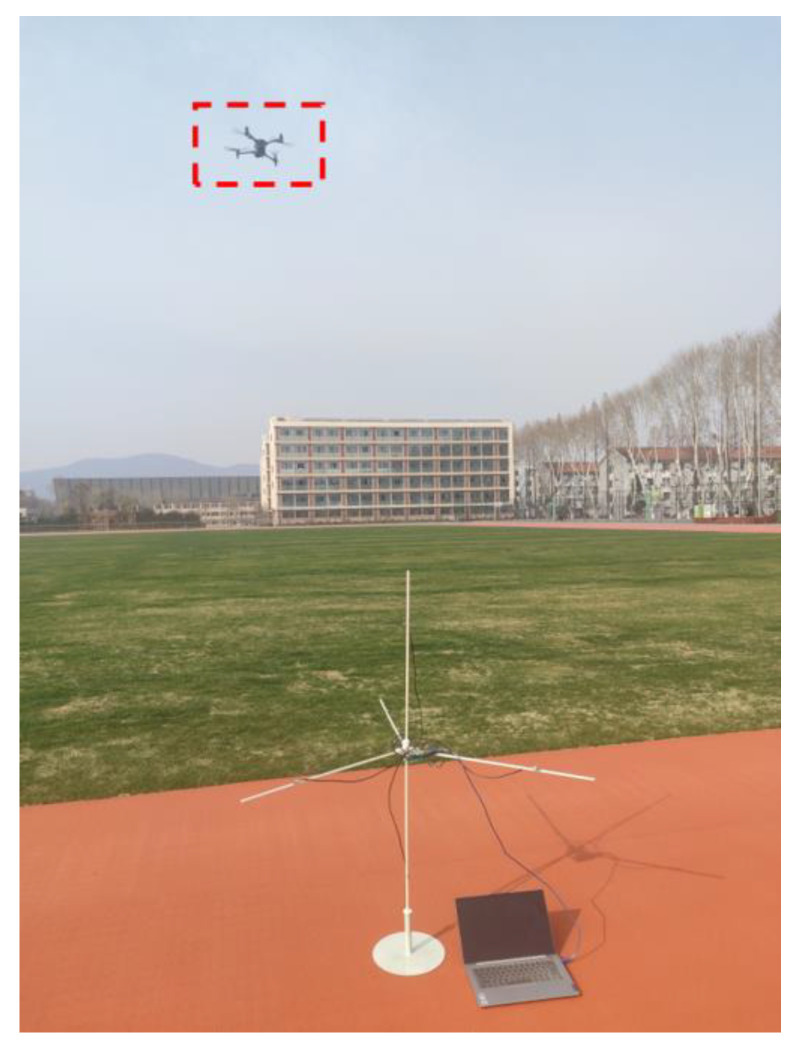
Collect data in a quiet environment.

**Figure 18:**
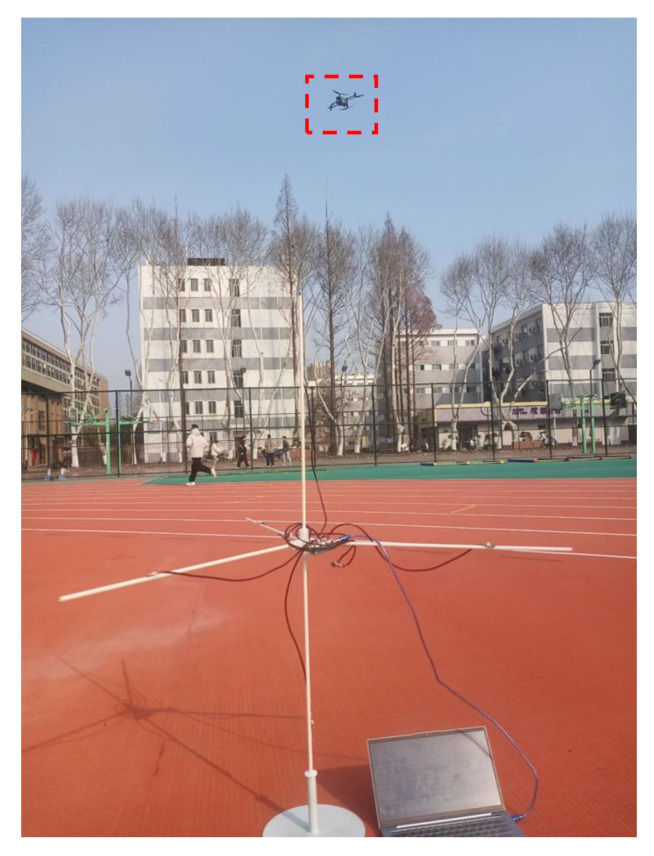
Collect data in a noisy environment.

This data collection is divided into two times, one at noon (the environment is relatively quiet), and the collection place is the middle of the playground, away from the road. Once, after school in the afternoon (the environment is noisy), the collection site is near the road and the basketball court. The data acquisition environment is shown in Figure 17 and Figure 18.

**Figure 19:**
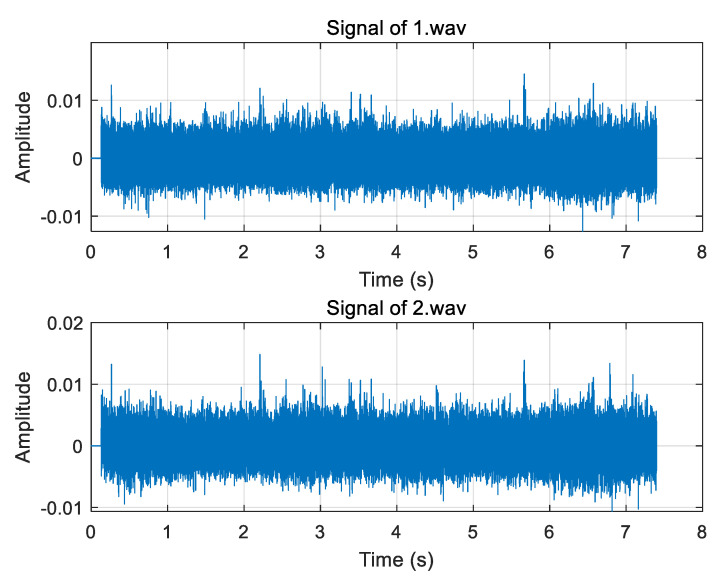
Picked up two drone sound signals.

Two of the UAV sound signals collected by the microphone array are shown in Figure 19. The normalization amplitude of the signal is about 0.01, which is easily disturbed by noise. In the improved weighting function, the parameter *α* is taken as 0.7, and the parameter *β* is taken as 0.01. Three groups of experimental data were collected, respectively, and the distance between the UAV and the microphone array center was 13.95 m, 14.64 m, and 19.18 m. Four algorithms (GCC PHAT, GCC SCOTg/PHAT, GSCC SCOTg/PHAT, and GTCC SCOTg/PHAT) were used to verify the time delay estimation. The experimental results are shown in Table 3 and Table 4.

As can be seen from Table 3 and Table 4, the *RMSE* of time delay estimation shows an increasing trend as the distance between the UAV and the array center increases. In a quiet environment, the *RMSE* of the four algorithms is less than 0.1 ms, the time delay estimation accuracy is higher, and the positioning error is less than 0.03 m. The accuracy of time delay estimation in a noisy environment decreases. When the distance is 19.18 m, the *RMSE* of the GCC PHAT algorithm, the GCC SCOTg/PHAT algorithm, and the GSCC SCOTg/PHAT algorithm is 0.00133 s, 0.00110 s, and 0.00088 s, respectively. The positioning errors are 45.22 cm, 37.40 cm, and 29.92 cm, respectively. The *RMSE* of the GTCC SCOTg/PHAT algorithm is 0.00065 s, and the positioning error is 22.10 cm, which is smaller than the error of the first two algorithms. Therefore, the GTCC SCOTg/PHAT algorithm has better noise resistance and effectiveness.

**Figure 20:**
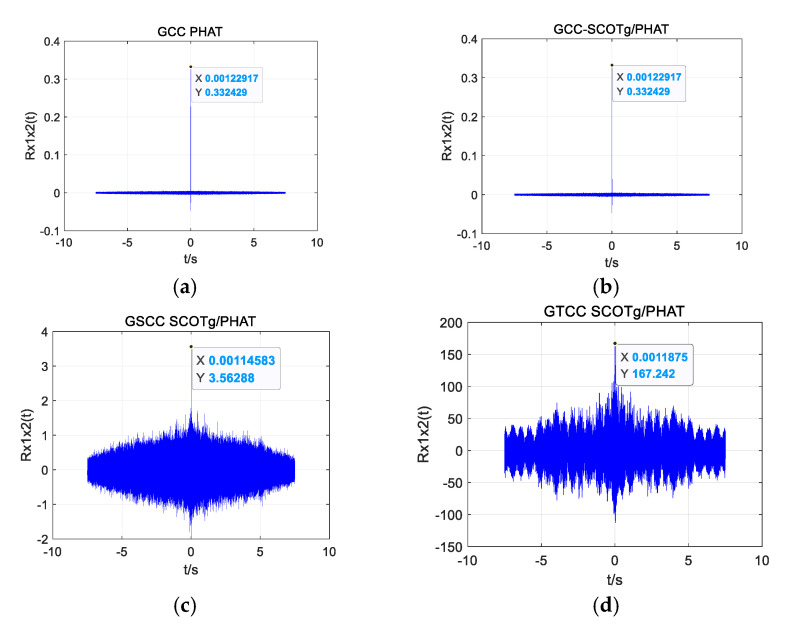
Time delay estimation of different algorithms in a quiet environment. (**a**) GCC PHAT. (**b**) GCC-SCOTg/PHAT. (**c**) GSCC SCOTg/PHAT. (**d**) GTCC SCOTg/PHAT.

**Figure 21:**
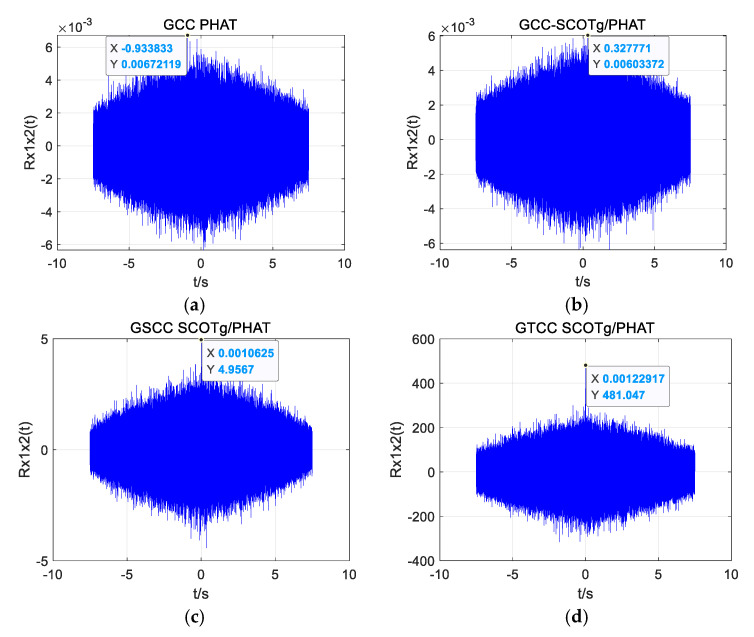
Time delay estimation of different algorithms in a noisy environment. (**a**) GCC PHAT. (**b**) GCC-SCOTg/PHAT. (**c**) GSCC SCOTg/PHAT. (**d**) GTCC SCOTg/PHAT.

When obtaining the data at a distance of 19.18 m as an example, the position coordinate of the UAV is (0, −12.2 m, 14.8 m), and the theoretical time delay between the UAV’s acoustic signal and the reference microphone 1 and microphone 2 is 0.0012 s. Figure 20a–c shows the time delay estimation of the four algorithms in a quiet environment. Time delay estimation of the four algorithms in a noisy environment is shown in Figure 21a–c.

As can be seen from Figure 20a–c and Figure 21a–c, all four algorithms can calculate relatively accurate time delay values in a quiet environment, among which the estimated time delay value of GTCC SCOTg/PHAT is 0.0011875 s, which is the smallest error among the four algorithms. But in a noisy environment, only the improved weighted function combined generalized cubic cross-correlation algorithm can calculate the more accurate time delay of 0.0012292 s. Although there are more sub-peaks in the GTCC SCOTg/PHAT algorithm compared with the other three algorithms in a quiet environment, due to the large difference between the main peak and the sub-peak, the correct main peak is not affected. In a noisy environment, the proposed algorithm has a significant cross-correlation peak without false peak interference. The false peaks produced by the cross-correlation function of the other three algorithms have exceeded the main peaks, which seriously affects the accuracy of time delay estimation.

## 6. Conclusions

In order to solve the problem of large errors in the time delay estimation algorithm at a low SNR, this paper introduces a time delay estimation algorithm based on an improved weighted function joint generalized cubic cross correlation. Through simulation and experiment, it is found that the improved weighted function combined with generalized cross-correlation operation can indeed reduce the interference of noise. By applying the improved weighting function to the cubic cross-correlation algorithm, the main peak value with the larger peak value can be obtained, and a more accurate time delay estimate can be calculated at a low SNR.

1. For the UAV sound signal, the optimal value of the exponential factor of the improved weighting function under the experimental conditions of this paper was studied. When the exponential factor *α* is not more than 0.7, the correct time delay can be obtained, and when *α* is 0.7, the main peak is the most prominent, and the noise suppression effect is the best. If the latency exceeds 0.7, the correct latency value cannot be obtained. Therefore, the extended estimation performance of the improved weighting function is the best when the exponential factor *α* is 0.7.
2. The simulation compares the time delay estimation performance of the traditional weighted function and the improved weighted function under different SNRs. The ROTH weighting function has the worst time delay estimation effect, and the performance of the SCOT and PHAT weighting functions is similar when the SNR drops to 0 dB. When the SNR drops to −5 dB, only the improved weighting function proposed in this paper can calculate a more accurate time delay value. Therefore, the improved weight function has better anti-noise performance than the traditional weight function.
3. The time delay estimation performance of the improved weighted function combined with the cross-correlation operation at different times is studied. At low signal-to-noise ratio (SNR), the improved weighted function combined generalized cubic cross-correlation algorithm has the highest correlation peak-to-peak value, which further improves the anti-noise performance of the algorithm. In the time delay estimation of the UAV acoustic signal detection in the real scene, the *RMSE* of different SNR algorithms is compared. The accuracy of the proposed algorithm is 35.55% higher than that of the traditional PHAT weighted algorithm and 22.78% higher than that of the improved weighted function combined first cross-correlation algorithm. GTCC SCOTg/PHAT algorithm can better meet the accuracy requirements of UAV detection in complex outdoor scenes.

In summary, the improved algorithm is helpful in improve the positioning ability of low-altitude UAVs and can provide a reference for signal processing of anti-low-altitude UAV combat systems and low-altitude UAV early warning systems.

## Figures and Tables

**Table 1:** Weighting functions.

Function Name	Weighting Function
Basic cross correlation	1
ROTH	$\frac{1}{G_{x_{1}x_{1}}\left(\omega \right)}\;or\;\frac{1}{G_{x_{2}x_{2}}\left(\omega \right)}$
SCOT	$\frac{1}{\sqrt{G_{x_{1}x_{1}}\left(\omega \right)G_{x_{2}x_{2}}\left(\omega \right)}}$
PHAT	$\frac{1}{\left|G_{x_{1}x_{2}}\left(\omega \right)\right|}$
ML	$\frac{|r_{x_{1}x_{2}}\left(\omega \right){|}^{2}}{G_{x_{1}x_{2}}\left(\omega \right)\left(1-|r_{x_{1}x_{2}}\left(\omega \right){|}^{2}\right)}$
HB	$\frac{\left|G_{x_{1}x_{2}}\left(\omega \right)\right|}{G_{x_{1}x_{1}}\left(\omega \right)G_{x_{2}x_{2}}\left(\omega \right)}$

included among these ${\left|r_{x_{1}x_{2}}\left(\omega \right)\right|}^{2}=\frac{|G_{x_{1}x_{2}}\left(\omega \right){|}^{2}}{G_{x_{1}x_{1}}\left(\omega \right)G_{x_{2}x_{2}}\left(\omega \right)}$.

**Table 2:** Performance parameters of the microphone acquisition device.

Name	Parameter
Directivity	omni directional
Sensitivity	−27~−25 dB
Operation voltage	1.6~3.6 V
Signal-to-noise ratio	61 dB
Current consumption	750 μA
Microphone board size	18 × 14 mm

**Table 3:** Comparison of the *RMSE* of four algorithms in a quiet environment.

Distance	GCC PHAT	GCC SCOTg/PHAT	GSCC SCOTg/PHAT	GTCC SCOTg/PHAT
13.95 m	0.000037 s	0.000032 s	0.000029 s	0.000017 s
14.64 m	0.000041 s	0.000035 s	0.000030 s	0.000028 s
19.18 m	0.000066 s	0.000061 s	0.000054 s	0.000043 s

**Table 4:** Comparison of *RMSE* of four algorithms in a noisy environment.

Distance	GCC PHAT	GCC SCOTg/PHAT	GSCC SCOTg/PHAT	GTCC SCOTg/PHAT
13.95 m	0.00053 s	0.00046 s	0.00050 s	0.00046 s
14.64 m	0.00081 s	0.00064 s	0.00063 s	0.00058 s
19.18 m	0.00133 s	0.00110 s	0.00088 s	0.00065 s

## Data Availability

Data on supporting conclusions have been charted and presented in this article.
